# The Complete Chloroplast Genome of *Heimia myrtifolia* and Comparative Analysis within Myrtales

**DOI:** 10.3390/molecules23040846

**Published:** 2018-04-08

**Authors:** Cuihua Gu, Bin Dong, Liang Xu, Luke R. Tembrock, Shaoyu Zheng, Zhiqiang Wu

**Affiliations:** 1 School of Landscape and Architecture, Zhejiang Agriculture and Forestry University, Hangzhou 311300, China; gucuihua@zafu.edu.cn (C.G.); 20170006@zafu.edu.cn (B.D.); zhengsy_gz@126.com (S.Z.); 2 Zhejiang Academy of Forestry, Hangzhou 310023, China; jachary@163.com; 3 Department of Biology, Colorado State University, Fort Collins, CO 80523, USA; Luke.Tembrock@colostate.edu; 4 Department of Ecology, Evolution and Organismal Biology, Iowa State University, Ames, IA 5011, USA

**Keywords:** *Heimia myrtifolia*, cp genome, codon usage, sequence divergence, phylogeny

## Abstract

*Heimia myrtifolia* is an important medicinal plant with several pharmacologically active alkaloids and is also used as an ornamental landscape plant. The purpose of this study is to complete and characterize the chloroplast (cp) genome of *H. myrtifolia* and compare genomic features to other Myrtales species’ cp genomes. The analysis showed that *H. myrtifolia* has a total length of 159,219 bp with a typical quadripartite structure containing two identical inverted repeats (IRs) of 25,643 bp isolated by one large single copy (LSC) of 88,571 bp and one small single copy (SSC) of 18,822 bp. The *H. myrtifolia* cp genome contains 129 genes with eight ribosomal RNAs, 30 transfer RNAs, and 78 protein coding genes, in which 17 genes are duplicated in two IR regions. The genome organization including gene type and number and guanine-cytosine (GC) content is analyzed among the 12 cp genomes in this study. Approximately 255 simple sequence repeats (SSRs) and 16 forward, two reverses, and two palindromic repeats were identified in the H. myrtifolia cp genome. By comparing the whole *H. myrtifolia* cp genome with 11 other Myrtales species, the results showed that the sequence similarity was high between coding regions while sequence divergence was high between intergenic regions. By employing the full cp genomes for phylogenetic analysis, structural and sequence differences were characterized between *H. myrtifolia* and 11 Myrtales species illustrating what patterns are common in the evolution of cp genomes within the Myrtales. The first entire cp genome in the genus *Heimia* provides a valuable resource for further studies in these medicinally and ornamentally important taxa.

## 1. Introduction

*Heimia* is a genus of flowering plants in the loosestrife family, Lythraceae (Order Myrtales), named in honor of German physician Ernst Ludwig Heim [1]. The genus *Heimia* is comprised of three woody shrub species with five-petaled yellow flowers and a bell-shaped or hemispherical calyx tube, and is commonly known as “sun opener” or “shrubby yellowcrest”. The *Heimia* species are distributed from west Texas and northern Mexico in the north to Argentina in the southern part of the range. *Heimia* species have a history of medicinal use in native American cultures, in which several pharmacologically active alkaloids have been found, chief among them being cryogenine [2,3]. *Heimia myrtifolia* has been reported to have hallucinogenic properties wherewith objects appear yellow accompanied with auditory hallucinations [3]. Anti-inflammatory properties have also been attributed to the alkaloid cryogenine in *Heimia* [4]. Given the attractive yellow flowers that *Heimia* species produce and its shrubby form, it is highly valued as ornamental plant.

Chloroplasts (cp), are essential organelles that convert light energy to chemical energy in chlorophytes and possess their own genomes for biosynthesis of pigments, starch, amino acids, and fatty acids, encoding proteins for photosynthesis and nitrogen fixation [5]. Compared with nuclear genomes, cp genomes have highly conserved gene order, number, and content, and are uniparentally inherited [6]. Most angiosperms’ cp genomes are typically circular with a quadripartite structure ranging from 115 to 165 kb in length and include two inverted repeated regions (IRs) which are separated by the small single copy region (SSC) and the large single copy region (LSC) [7]. Because of their conserved structure, uniparental inheritance, and similar gene content, DNA sequences from cp genomes have been important in systematic, population genetic, and phylogenetic studies. Previously, phylogenetic trees have been reconstructed from one or a few genes from the cp [8]. However, in recent years, complete cp genomes have been increasingly used as an informative resource for resolving lower taxonomic level phylogenetic relationships [9,10,11,12,13,14,15].

By comparing entire cp genomes, the ability to detect reliable DNA barcodes for precise plant identification is improved. As next-generation sequencing costs fall, cp genomes are more routinely integrated into phylogenetic, population genetics, and DNA barcoding for identification of numerous species and families [9,10,13,16,17,18,19,20,21]. The over 2300 cp genomes that have been deposited in the National Center for Biotechnology Information (NCBI) database illustrates the importance and utility of whole cp genomes for the study of plant evolution.

Herein, we present the first whole cp genome sequence generated from Illumina sequencing in the genus *Heimia*. This complete cp genome will be a valuable genetic resource for comprehensively understanding the organization of the *H. myrtifolia* cp genome and studying phylogenetic relationships within the Lythraceae family and Myrtales generally. Our study objectives were as follows: to enhance our understanding of the structural diversity of the *H. myrtifolia* genome and detect highly informative hotspot markers from comparative analyses with other cp genomes in Lythraceae and Myrtales.

## 2. Results and Discussion

### 2.1. Chloroplast Genome Structure and Content

**Figure 1:**
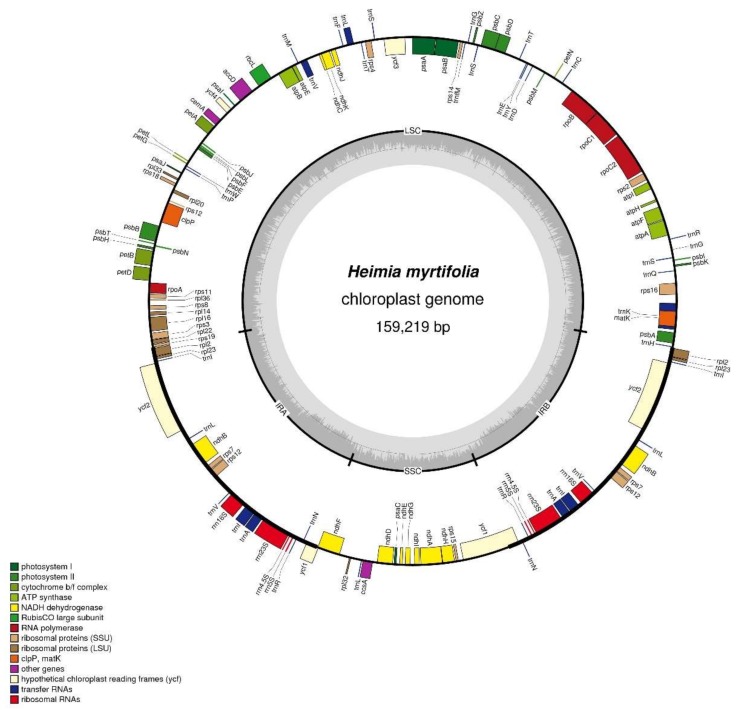
Structural map of the *Heimia myrtifolia* cp (chloroplast) genome. The map is a quadripartite and circular structure which was drawn by OGDRAW. Genes of different functional groups are separated by color. The innermost grey region inside the inner circle refers to percent GC content in this cp genome. Genes shown outside and inside of the outer circle are transcribed counterclockwise and clockwise, respectively (LSC: Large single-copy region; IR: Inverted repeat; SSC: Small single-copy region).

The *H. myrtifolia* cp genome is 159,219 bp (Figure 1) in length and similar to other Myrtales cp genomes (Table 1 and Table 2), which vary in length from 152 to 165 Kb [20,22]. Unsurprisingly, the cp DNA of *H. myrtifolia* is the typical quadripartite and circular structure that contains two IRs divided by LSC and SSC regions (Figure 1). The guanine-cytosine (GC) content percentage of the intact *H. myrtifolia* cp genome was 37.0% (Table 1), which is lower than that of *L. intermedia* (37.6%) and *Oenothera argillicola* (39.1%).

In the *H. myrtifolia* cp genome, 112 total unique genes were detected, of which 17 are duplicated in the IRs (Table 3). The 112 genes are divided into 30 tRNA genes, four rRNA genes, and 78 protein-coding genes. Among these 112 unique genes, three (*clpP*, *rps12*, and *ycf3*) contain two introns and 14 contain one intron (eight protein-coding genes and six tRNA genes) (Table 4). The *Rps12* gene is a trans-spliced with two C-terminal exons and one N-terminal downstream exon. The *trnK*-UUU gene in which the *matK* gene is located has the largest intron at 2497 bp.

By proportion, tRNAs, rRNAs, and proteins are encoded by 2.0, 3.0, and 51.0% of the *H. myrtifolia* cp genome, respectively (Table 2). The remaining 49.0% of the *H. myrtifolia* cp genome belongs to non-coding regions, comprised of pseudo-genes, introns, and intergenic spacers (Table 2). Protein-coding sequences account for 74,088 bp possessing 78 protein-coding genes coding for 27,453 codons (Table 3 and Table S1). Moreover, the AT content within protein-coding regions was 66.1%, 61.9%, and 58.7% at the first, second, and third codon positions, respectively (Table 5). At the third codon position, G and C nucleotides are enriched over A and T; a result consistent with those widely obtained in many other terrestrial plant cp genomes [23].

### 2.2. Codon Usage

Codon usage biases can have important ramifications for cellular function and reflect lineage specific translational systems thus providing additional means for studying speciation and evolution at the molecular level [24,25]. However, cp genomes, unlike nuclear genomes, do not appear to have synonymous codon usage bias associated with intron number or evolutionary specialization [26]; therefore, we examined codon usage to confirm this.

**Figure 2:**
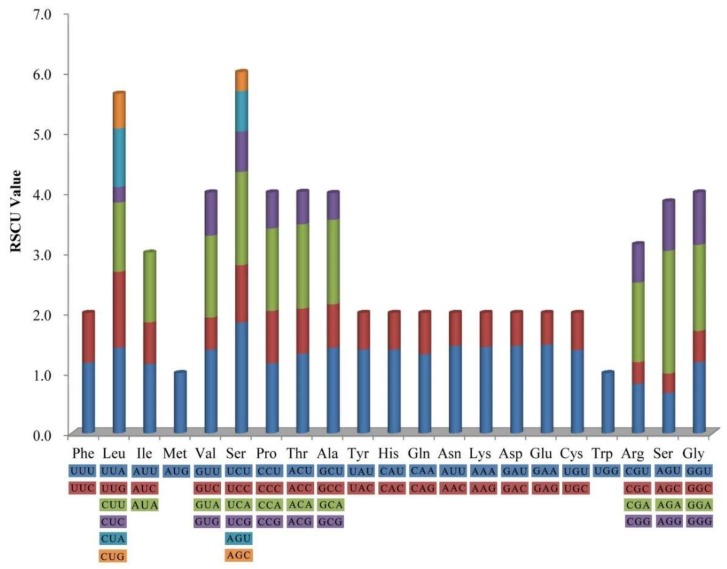
Codon content of 20 amino acids and stop codon including all 78 protein-coding genes in *H. myrtifolia* cp genome. The color of codons corresponds to color of the histogram.

The frequency of codon usage was calculated for the *H. myrtifolia* cp genome based on the tRNAs and protein-coding genes. Tryptophan (1.5%) and leucine (11.6%) were the least-frequency and highest-frequency amino acids, respectively (Figure 2). Among which, the least and most used were CGC (99) encoded arginine and AAA (1137) encoded lysine, respectively (Table S1). Significantly, as a synonym, almost each amino acid contains half of the codons, which ended with A or T (U) at high relative synonymous codon usage (RSCU) values and low RSCU values ended with G or C (Table S1). The composition bias with high A/T proportion codon usage patterns is generally semblable to those reported from other cp genomes [27].

### 2.3. Comparative Genomic Analysis of the cp Genomes in Myrtales

**Figure 3:**
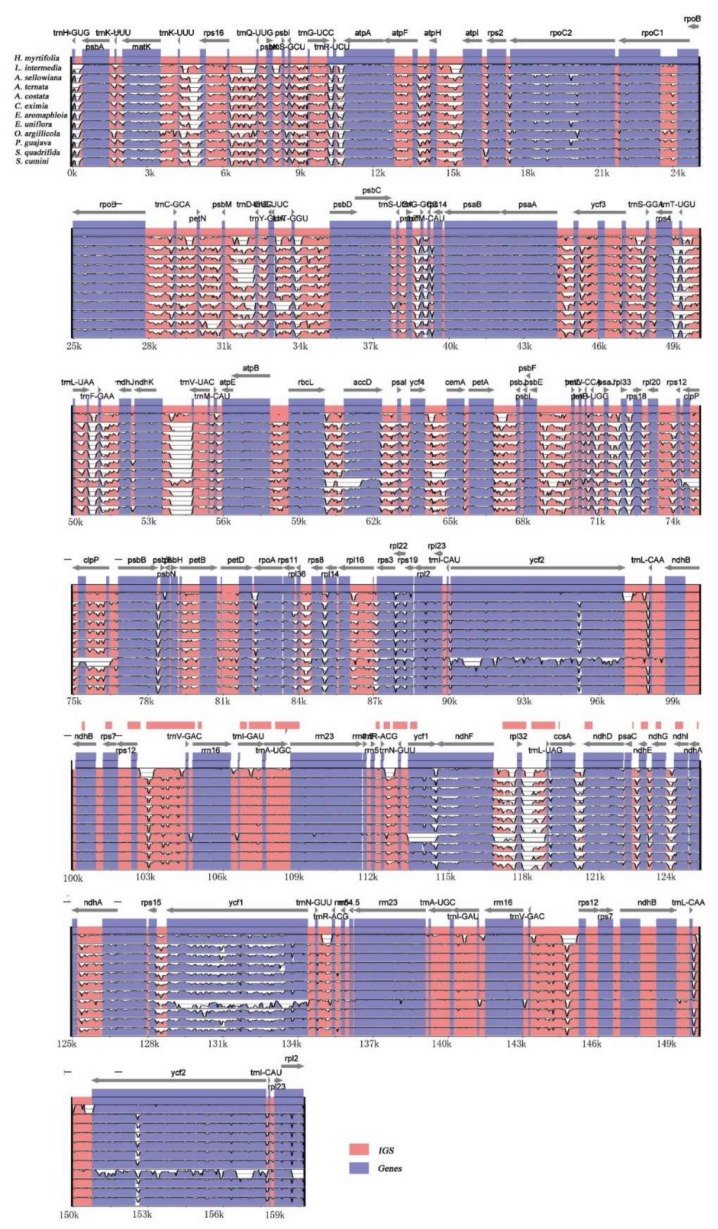
Visualization alignments among the 12 Myrtales cp genomes. VISTA-based identity plot showing sequence identity using *H. myrtifolia* as reference. The *y*-axis indicates % identity ranging from 50 to 100% to the reference. Protein-coding genes and intergenic regions are marked in purple and pink, respectively.

From the pairwise comparison of cp genomes, a high level of sequence similarity was found between *H. myrtifolia* and the 11 other Myrtales cp genomes. By using mVISTA, *H. myrtifolia* annotation was used as a reference to characterize differences between the 11 Myrtales species’ cp genomes (Figure 3). The results showed that the LSC and SSC regions are more divergent than the two IR regions. In addition, within the LSC and SSC regions, the non-coding regions are more divergent than the coding regions. The most highly differentiated regions including *atpB, matK*, *ndhD*, *ndhF*, *ndhH*, *rpl22*, *rps15*, *ycf2*, and *trnH-psbA*. Similar levels of divergence have been previously measured for these gene regions [28,29]. IR regions of all 12 cp genomes were highly conserved, including gene order and number, however, they showed significant differences at the junction of the single-copy regions. Neither inversions nor translocations were detected among these compared genomes. Variations of genome size, IR expansion, and contraction were the main structural differences detected within these 12 cp genomes.

#### 2.3.1. Genome Size Differences between the 12 Myrtales cp Genomes

For genome size of the 12 Myrtales species examined, *L. intermedia* has the smallest cp genome size (152,330 bp) and *Oenothera argillicola* the largest (165,055 bp). The genome size variation is largely caused by differences in the intergenic regions (IGS), similar to other angiosperm cp genomes.

#### 2.3.2. Contraction and Expansion of All Inverted Repeats (IRs)

**Figure 4:**
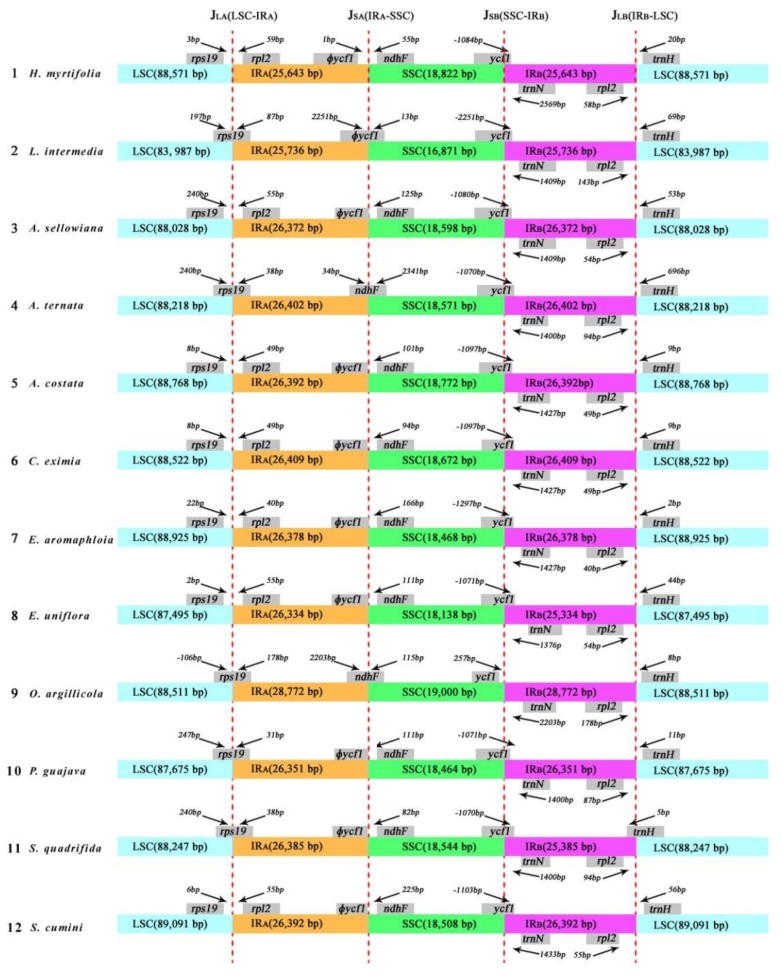
The comparison of the LSC, IRs, and SSC junction boundaries among 12 species cp genomes. Boxes above or below the main line indicate the adjacent border genes. Number in bp marked above indicates the gap between the ends of the boundaries and adjacent genes (these features are not to scale). The ψ notation indicates pseudogene.

In general, the sizes of IR regions differ between species (Table 1). The expansion and contraction between the two inverted repeats, LSC, and SSC boundary regions usually generates length variation of plant cp genomes [30]. Accurate SC–IR boundaries and their neighboring genes were compared among the 12 Myrtales cp genomes (Figure 4). Although the overall genomic structure was conserved, the 12 Myrtales cp genomes possessed differences at the SC–IR junction regions (Figure 4).

The size of two IRs varied from 25,736 bp (*L. intermedia*) to 28,772 bp (*O. argillicola*), as did the four IR boundaries (J_LA_, J_LB_, J_SA_, and J_SB_) [13] (Figure 4). The IR_A_–LSC boundary (J_LA_) is nested in the *rps19* coding gene in *L. intermedia*, *A. ternata*, *O. argillicola*, *P. guajava*, and *S. quadrifida* by 87 bp, 38 bp, 178 bp, 31 bp, and 38 bp, respectively, into the IR_A_ region. However, in the remaining seven species, the J_LA_ boundary nested in the intergenic region between *rps19* and *rpl2*, in which the distances from *rps19* to the J_LA_ ranged from 2 to 240 bp. The IR_A_–SSC junction (J_SA_)is nested in the pseudogene *ycf1* (*ϕycf1*) in *L. intermedia* (Figure 4). The J_SA_ junction for eight of the 12 species (*A. sellowiana*, *A. costata, C. eximia, E. aromaphloia, E. uniflora, Psidium guajava, S. quadrifida*, and *S. cumini*) is located on the edge of *ϕycf1*. The J_SA_ junction of *A. ternata* and *O. argillicola* was located in the range of *ndhF*, and J_SA_ of *H. myrtifolia* is situated 1 bp from the end of *ϕycf1*.

The IR_B_–SSC boundary (J_SB_) in 11 of the 12 species is nested in the *ycf1* gene, which extended into IR_B_ region, while in *O. argillicola*, the distance between J_SB_ and the end edge of *ycf1* was 257 bp. The IR_B_–LSC boundary (J_LB_) was situated in the region between *rpl2* and *trnH* in all of the species except *S. quadrifida.* In *S. quadrifida*, the *trnH* gene extends 5 bp into IR_B_ (Figure 4). The IR-LSC boundary variation is likely the result of a series of two short direct repeats that are mediated by intramolecular recombination within the genes located at the borders [31]. As such, the IR–LSC boundary could be a highly informative region for population or phylogenetic studies.

#### 2.3.3. Long Repeat Structure Analysis

Previous studies have shown that the genome rearrangement can occur from sliding and inappropriate combinations of repetitive sequences [32]. Long repetitive sequences have been highly valuable markers in the study of plant evolution, genome recombination studies, comparative genomics, and phylogenetics [33].

Comparison of forward, reverse, complement, and palindromic repeats (≥30 bp) were made among *H. myrtifolia* and 11 species using REPuter. In *H. myrtifolia*, 18 repeats including 15 forward, one palindromic, and two reverse type were found. *A. ternata* had the fewest (11) repeats with shortest genome size of 159,593 bp, which is inconsistent strictly with the rule of larger genome size possessing more repetitive repeats [34].

**Figure 5:**
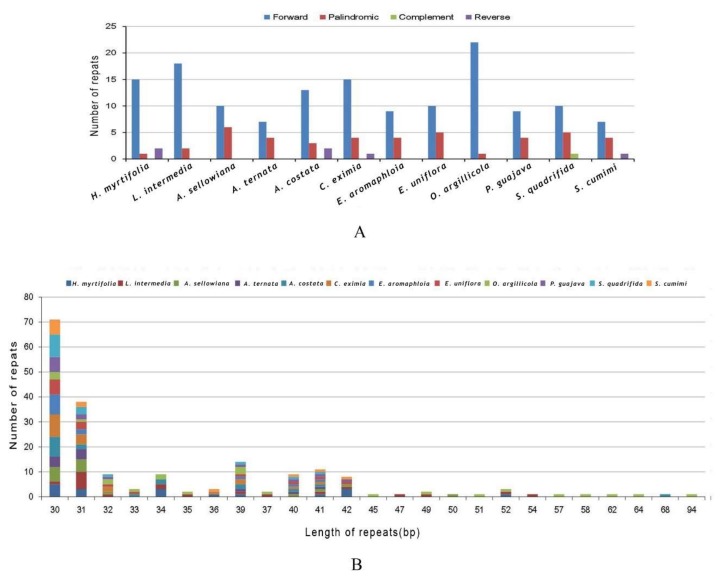
Number of long repetitive repeats in 12 Myrtales complete cp genomes. (**A**) Frequency of repeat types; (**B**) Frequency of the repeats more than 30 bp long.

In total, 195 repeats in all 12 species were found (Figure 5A). *O. argillicola* possessed the greatest number of repeats consisting of 22 forward repeats and one palindromic repeat as well as possessing the largest genome of those in this study (Figure 5A and Table S2). In *L. intermedia, A. sellowiana, A. costata, C. eximia, E. aromaphloia, E. uniflora, P. guajava, S. quadrifida*, and *S. cumini* cp genomes, 20, 16, 18, 20, 13, 15, 13, 16, and 12 long repeats were identified, respectively (Figure 5A). The largest proportion of repeats (82.1%) varied from 30 bp to 40 bp in length (Figure 5B and Table S2), while the range of repeats was from 94 bp to 30 bp per unit. Forward repeats are usually caused by transposon activity [35], which can correlate with enhanced cellular stress [36]. Forward repeats can cause variation in genome structure and consequently can be employed as markers in population genetic and phylogenetic studies [20].

#### 2.3.4. Simple Sequence Repeat (SSR) Analysis

Simple sequence repeats (SSRs) in cp genomes have high copy number diversity and are thus very useful molecular markers for plant population genetics, breeding studies at the intraspecific level and evolutionary research [37]. In this study, the type, distribution, and number of SSRs were identified using the search criteria as follows: 10 repeats for mononucleotide, three repeats for dinucleotide, trinucleotide, tetranucleotide, pentanucleotide, and hexanucleotide among the cp genomes of 12 species.

**Figure 6:**
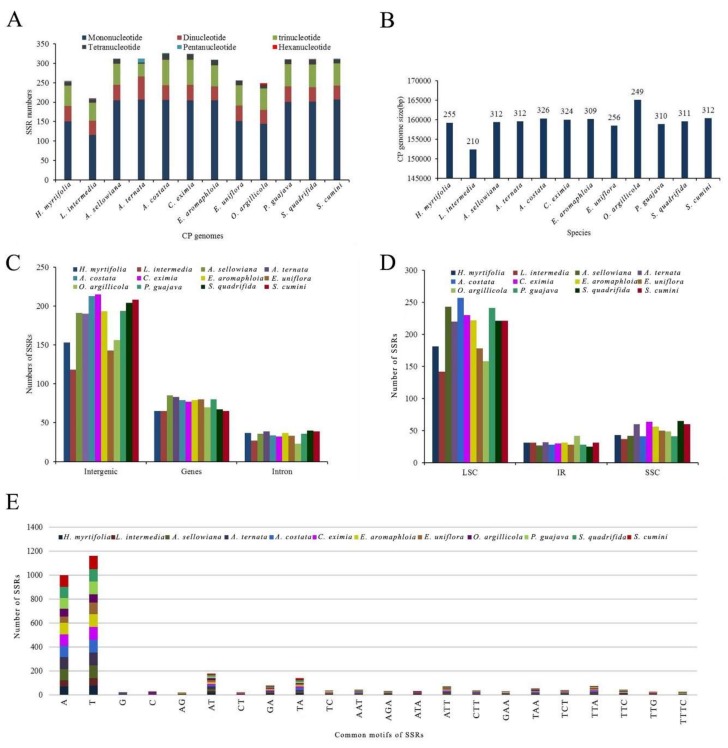
The comparison of simple sequence repeats (SSRs) distribution in 12 cp genomes. (**A**) Number of different SSR types detected in 12 chloroplast genomes; (**B**) Relationship between total SSRs number and the length of 12 cp genomes; (**C**) Frequency of SSRs in the intergenic regions, protein-coding genes and introns; (**D**) Frequency of SSRs in the LSC, IR, and SSC regions; (**E**) Frequency of common motifs in the 12 cp genomes.

Through SSRHunter analysis, 12 cp genomes were found to contain 210–326 SSRs (*H. myrtifolia*: 255, *L. intermedia*: 210, *A. sellowiana*: 312, *A. ternata*: 312, *A. costata*: 326, *C. eximia*: 324, *E. aromaphloia*: 309, *E. uniflora*: 256, *O. argillicola*: 249, *P. guajava*: 310, *S. quadrifida*: 311, and *S. cumini*: 312) (Figure 6A,B and Table S3). Among the 12 species, *L. intermedia* had the fewest 210 SSRs (Figure 6A) as well as the shortest cp genome (152,330 bp) among those studied. This suggests that the number of SSRs in these species may have some correlation with the genome size.

Among SSRs found herein, the mononucleotide repeat units A/T and G/C with repeat number from eight to 18 accounted for the largest proportion with 66.4% in *A. ternata* and *S. cumini*, 66.3% in *E. aromaphloia*, 65.7% in *A. sellowiana,* 64.9% in *S. quadrifida*, 64.8% in *P. guajava*, 63.2% in *C. eximia*, 63.1% in *A. costata*, 59.4% in *E. uniflora*, 59.2% in *H. myrtifolia*, 57.8% in *O. argillicola*, and 55.2% in *L. intermedia* (Figure 6A and Table S3). Among the 255 SSRs in *H. myrtifolia*, 153 SSRs were found in intergenic regions (IGS), 65 SSRs in protein-coding regions, and 37 SSRs in introns (Figure 6C,D). The higher number of SSRs in the IGS regions might be contributing to the increased mutation rates in these regions over coding regions, given the higher rate of SSR mutation. In the *H. myrtifolia* cp genome, 65 SSRs were situated in 28 protein-coding genes (*ycf1* (10), *ycf2* (14), *ndhD, petA, psbB, psbE, rbcL, rpoC2* (4), *ndhF* (3), *atpB*, *atpI*, ccsA, *cemA*, *matK*, *ndhA*, *ndhB*, *ndhK*, *psaA*, *psaB*, *psaJ*, *rpl2*, *rpl22*, *rpl32*, *rpoA*, *rpoB*, *rpoC1*, *rps19* (2)*, ycf4*). In general, the cp genomes examined had an abundant diversity of SSRs for use in future studies.

#### 2.3.5. Divergence Hotspots among Myrtales Species

**Figure 7:**
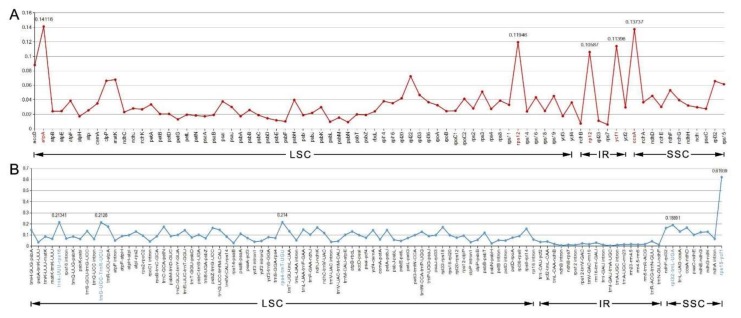
The nucleotide variability (Pi) value in the 12 aligned Myrtales chloroplast genomes. (**A**) Protein-coding genes (the five genes marked in red are the highest five in all genes). (**B**) Intergenic regions. These regions are oriented according to their locations in the chloroplast genome (the five regions marked in blue are the highest five in intergenic regions).

The nucleotide diversity (Pi) values of the 12 species’ cp genomes were computed separately for the IRs, LSC, SSC regions, and protein-coding genes including introns (Figure 7A,B). The IGS regions were far more divergent than the protein-coding regions (CDS). In regard to the quadripartite subdivisions, the LSC and SSC are less divergent than IRs regions. Within the CDS regions, Pi values varied from 0.09 to 0.141 with an average value of 0.033 in the LSC region, the SSC region ranged from 0.028 to 0.137, with an average value of 0.051, and the IR region had values from 0.005 to 0.114 with an average value of 0.046.

The five genes with the largest variability in CDS region were *atpA*, *ccsA*, *rps12*, *ycf1*, and *rpl2* (Figure 7A), and for the IGS regions, *rps15-ycf1, rps4-trnT-UGU, trnK-UUU-rps16, trnG-UCC-trnR-UCU,* and *rpl32-trnL-UAG* were the most variable (Figure 7B). Some regions were uncharacteristically conserved with IGS regions *trnI-GAU-trnA-UGC* and the *ndhB* intron showing less variation than that of genes situated in the CDS region (Figure 7B).

#### 2.3.6. Phylogenetic Analysis of *H. myrtifolia* and Related Myrtales cp Genomes

**Figure 8:**
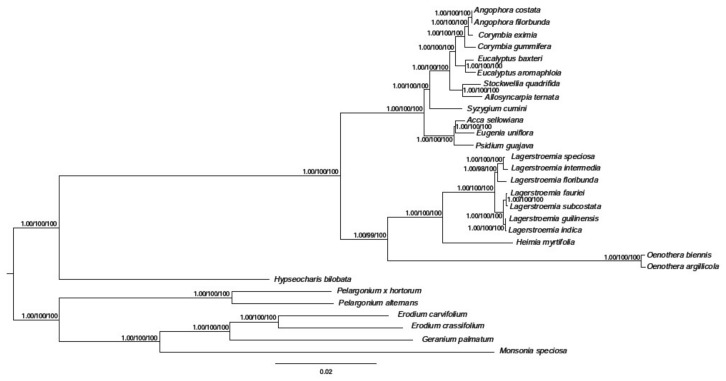
Phylogenetic tree based on 68 shared protein-coding genes was constructed for 29 species using three different methods, including Bayesian inference (BI), Parsimony analysis (MP), and Maximum likelihood (ML). The posterior probability or bootstrap values as 1.0 or 100 were not shown on the nodes of tree, only the values lower than 1.0 or 100 were shown for each method respectively.

In the past few decades, the method of constructing phylogenetic trees has been based on one or a few relatively short sequences [38]. However, due to lateral gene transfer, paralogy, and genetic evolution rate differences between groups, the phylogenetic tree based on a single or few genes cannot sufficiently represent phylogenetic relationships. The entire cp genome is being used more and more in plant phylogenetic and population genetics as large-scale DNA sequencing becomes more main stream and less expensive. Our phylogenetic tree showed that *H. myrtifolia* is most closely related to *Lagerstroemia* species based on the 68 shared protein-coding genes in the matrix (Figure 8). Through all three methods, the phylogenetic tree had very high bootstrap support for most branches. These results suggested that entire cp genome information may be useful when resolving phylogenetic relationship conflicts. However, phylogenetic analyses with many closely related species and populations are needed to thoroughly examine the resolving power of cp coding genes [13,39].

## 3. Materials and Methods

### 3.1. DNA Extraction of Plant Materials and Sequencing

Fresh leaves of *H. myrtifolia* (Lythraceae, Myrtales) were attained from Hangzhou Botanic Garden, Zhejiang Province (China), and were preserved immediately in silica gel. Genomic DNA was extracted employing a standard Cetyl trimethyl ammonium bromide (CTAB) protocol [40]. The concentration and quality of extracted DNA was evaluated using a NanoDrop 2000 Micro spectrophotometer and an Agilent 2100 Bioanalyzer (Agilent Technologies, Santa Clara, CA, USA).

A sequence library was constructed using purified DNA following the manufacturer’s instructions. Using an Illumina HiSeq 2000 sequencer (Illumina Biotechnology company, San Diego, CA, USA), approximately 41,103,536 raw reads were obtained with paired-end (PE) 150 bp length reads.

### 3.2. Chloroplast Genome Assembly, Annotation, and Structure

Using Trimmomatic v0.3, raw reads with a Phred Quality Score of 20 or less were trimmed and filtered [41] using the following settings: sliding window: 4:15, trailing: 3, leading: 3, and minlen: 50. First, the CLC Genomics Workbench v7.0 (Qiagen Company, Hilden, Germany) was employed to carry out *de novo* assembly with the default parameters [13]. Second, using the *Lagerstroemia fauriei* cp genome as a reference, all contigs were aligned using BLAST software on the NCBI website to generate the complete cp genome.

Genome annotation was performed for the ribosomal RNAs (rRNAs), transfer RNAs (tRNAs), and protein-coding genes using DOGMA v1.2 [42]. The start and stop codons and the exon–intron boundaries of genes were precisely manually confirmed using published cp genomes [39]. Draft annotations were subsequently examined and manual adjustments were made with alignments to related species *L. fauriei* [13]. BLASTN searches in the NCBI website were used to identify and confirm both tRNA and rRNA genes. Lastly, further verification of the tRNA genes was carried out with tRNAscan-SE v1.21 [43]. The final cp genome physical map was drawn using OGDraw software [44].

### 3.3. Codon Usage

In order to detect the deviation in the use of synonymous codons, the relative synonymous codon usage (RSCU) was used to examine the effect of amino acid composition as calculated by MEGA 6 [45]. The RSCU is a simple method to determine synonymous codon inconsistencies in coding sequences. The RSCU value is the relative probability for a specific codon when translating the corresponding amino acid and it removes the effect of the amino acid composition on the use of the codon. An RSCU of >1.00 denotes codons are used more frequently than expected, while an RSCU of <1.00 denotes a codon is being applied less frequently than expected.

### 3.4. Genome Comparative Analysis and Molecular Marker Identification

We downloaded *Lagerstroemia intermedia*, *Acca sellowiana*, *Angophora costata*, *Allosyncarpia ternata*, *Corymbia eximia*, *Eucalyptus aromaphloia*, *Eugenia uniflora*, *Oenothera argillicola*, *Psidium guajava*, *Syzygium cumini*, and *Stockwellia quadrifida* cp genomes from GenBank (GenBank accession numbers in Table 1 and Table 2), as a set to compare cp genomes in the Myrtales. Using the annotation of H. myrtifolia as the reference, pairwise alignments among 12 cp genomes in the Myrtales were conducted using LAGAN mode in the mVISTA program [46].

In order to assess the different evolutionary patterns in Myrtales and detect the highly informative regions, we extracted both intergenic regions and protein-coding regions after alignment using MEGA 6. The two-standard cutoff was used wherein at least one mutation site must be present and the aligned length is >200 bp. The nucleotide diversity (Pi) of these regions was calculated using DNaSP V5.10 [47].

### 3.5. IR Expansion and Contraction of cp Boundaries

Genome differences between species are often found at the LSC and SSC junctions with the two reverse duplicate regions (IR_A_ and IR_B_). There are four boundaries (JL_A_, JL_B_, JS_A_, and JS_B_) in the cp genome between the two IRs and the LSC and SSC regions [30]. The precise IR expansion and contraction with the boundary genes among *H. myrtifolia* and the 11 other Myrtales species were compared in this study.

### 3.6. Identification of Long Repetitive Sequences and Simple Sequence Repeats (SSRs)

Long repetitive repeat sequences, including forward, reverse, palindromic, and complement repeats, were identified by employing REPuter [48]. The settings for identifying long repetitive repeats were used as follows: (1) a minimum repeat size of 30 bp; (2) 90% or greater sequence identity; (3) a Hamming distance of 3 [49]. To find SSRs within the cp genome, SSRHunter was employed using the following parameter settings for each motif type: mononucleotides ≥ 8; dinucleotides ≥ 4; trinucleotides, tetranucleotides, pentanucleotide, and hexanucleotide SSRs ≥ 3.

### 3.7. Phylogenetic Analysis

To analyze the phylogenetic placement of *H. myrtifolia*, 68 common protein-coding genes of the cp genomes from 29 species were employed including 6 outgroup species from Geraniaceae (*Erodium carvifolium*, *Erodium crassifolium*, *Monsonia speciosa*, *Pelargonium alternans*, *Pelargonium* x *hortorum*, and *Geranium palmatum* (GenBank accession numbers of species in Table S5). With the Clustal X default parameters, alignments were conducted to retain the reading frames accompanied by manual correction [50]. The data matrix used in the phylogenetic analyses is attached as supplemental data (Supplementary Materials). The phylogenetic tree based on these 68 concatenated genes was constructed using three phylogenetic-inference methods: maximum-likelihood (ML) using PHYML v 2.4.5 [51], Bayesian inference (BI) using MrBayes 3.1.2 [52] and parsimony analysis using PAUP* 4.0b10 [53] employing the settings from [13].

## 4. Conclusions

By adopting high coverage Illumina sequencing, we completed the *H. myrtifolia* cp genome and deposited the sequence into GenBank (Accession number: MG921615). The general genome structure, gene number, and gene content of *H. myrtifolia* were similar with all other cp genomes from Myrtales. However, numerous differences were found between the 12 species examined that are useful markers for studies in molecular evolution of cp genomes. The cp genome information of *H. myrtifolia* is a useful genetic resource that could be applied to population genomic studies for Lythraceae species and help elucidate genomic patterns and the evolutionary history in the group more broadly.

## Figures and Tables

**Table 1:** Summary of complete chloroplast genomes for *Heimia myrtifolia* and 11 other species in Myrtales.

	*H. myrtifolia*	*L. intermedia*	*A. sellowiana*	*A. ternata*	*A. costata*	*C. eximia*	*E. aromaphloia*	*E. uniflora*	*O. argillicola*	*P. guajava*	*S. quadrifida*	*S. cumini*
Accession Number	MG921615	NC034662	KX289887	KC180806	NC022412	NC022409	NC022396	NC027744	EU262887	NC033355	NC022414	GQ870669
Family	Lythraceae	Lythraceae	Myrtaceae	Myrtaceae	Myrtaceae	Myrtaceae	Myrtaceae	Myrtaceae	Onagraceae	Myrtaceae	Myrtaceae	Myrtaceae
Total length (bp)	159,219	152,330	159,370	159,593	160,326	160,012	160,149	158,445	165,055	158,841	159,561	160,373
guanine-cytosine (GC) (%)	37.0	37.6	37.0	37.0	37.0	37.0	37.0	37.0	37.0	39.1	37.0	37.0
**LSC**												
Length (bp)	88,571	83,987	88,028	88,218	88,768	88,522	88,925	87,459	88,511	87,675	88,247	89,091
GC (%)	35.0	36.0	35.0	35.0	35.0	35.0	35.0	35.0	37.0	35.0	35.0	35.0
Length (%)	55.6	55.1	55.2	55.3	55.4	55.3	55.5	55.2	53.6	55.2	55.3	55.6
**SSC**												
Length (bp)	18,822	16,871	18,598	18,571	18,772	18,672	18,468	18,138	19,000	18,464	18,544	18,508
GC (%)	30.6	30.9	31.0	31.0	30.0	31.0	31.0	31.0	35.0	31.0	31.0	31.0
Length (%)	11.8	11.1	11.7	11.6	11.7	11.7	11.5	11.4	12.0	12.0	12.0	12.0
**IRs**												
Length (bp)	25,643	25,736	26,372	26,402	26,392	26,409	26,378	26,334	28,772	26,351	26,385	26,392
GC (%)	42.6	42.5	43.0	43.0	43.0	43.0	43.0	43.0	43.0	43.0	43.0	43.0
Length (%)	16.1	16.9	16.5	16.5	16.5	16.5	16.5	16.6	35.0	35.0	33.0	33.0

LSC, large single-copy region; SSC, short single-copy region; IRs, inverted repeats.

**Table 2:** Distribution of genes and Intergenic regions for *Heimia myrtifolia* and 11 other species in Myrtales.

	*H. myrtifolia*	*L. intermedia*	*A. sellowiana*	*A. ternata*	*A. costata*	*C. eximia*	*E. aromaphloia*	*E. uniflora*	*O. argillicola*	*P. guajava*	*S. quadrifida*	*S.cumini*
Accession Number	MG921615	NC034662	KX289887	KC180806	NC022412	NC022409	NC022396	NC027744	EU262887	NC033355	NC022414	GQ870669
Family	Lythraceae	Lythraceae	Myrtaceae	Myrtaceae	Myrtaceae	Myrtaceae	Myrtaceae	Myrtaceae	Onagraceae	Myrtaceae	Myrtaceae	Myrtaceae
**Protein Coding Genes**												
Length (bp)	81,047	78,749	78,576	78,693	68,257	68,889	68,085	78,777	70,706	78,410	68,746	68,448
GC (%)	37.0	43.0	38.0	38.0	43.0	43.0	43.0	38.0	43.0	38.0	43.0	43.0
Length (%)	51.0	52.0	49.0	49.0	43.0	43.0	43.0	50.0	43.0	49.0	43.0	43.0
**rRNA**												
Length (bp)	9050	9050	9060	9056	9020	9056	9056	9050	9102	9056	9056	9050
GC (%)	55.0	55.0	55.0	55.0	55.0	55.0	55.0	55.0	55.0	55.0	55.0	55.0
Length (%)	3.0	3.0	3.0	3.0	3.0	3.0	3.0	3.0	3.0	3.0	3.0	3.0
**tRNA**												
Length (bp)	2817	2813	2779	2716	2184	2199	2270	2792	2303	2790	2387	2310
GC (%)	53.0	53.0	52.0	52.0	49.0	53.0	53.0	52.0	53.0	52.0	52.0	53.0
Length (%)	2.0	2.0	2.0	2.0	1.0	1.0	1.0	2.0	1.0	2.0	1.0	1.0
**Intergenic Regions**												
Length (bp)	50,172	46,156	51,541	51,503	65,351	64,369	65,018	49,679	69,633	50,496	63,907	65,069
GC (%)	32.0	33.0	35.0	35.0	35.0	35.0	35.0	35.0	37.0	35.0	35.0	35.0
Length (%)	32.0	30.0	32.0	32.0	41.0	40.0	41.0	31.0	42.0	32.0	40.0	41.0
**Intron**												
Length (bp)	16,133	15,562	17,414	17,625	15,514	15,499	14,720	18,147	13,311	18,089	15,465	15,496
GC (%)	38.0	37.0	37.0	37.0	35.0	36.0	36.0	37.0	38.0	38.0	36.0	36.0
Length (%)	10.0	10.0	11.0	11.0	10.0	10.0	9.0	11.0	8.0	11.0	10.0	10.0

**Table 3:** Genes in the sequenced *Heimia myrtifolia* chloroplast genome.

Category of Genes	Function of Genes	Name of Genes
Subunits of ATP synthase	Genes for photosynthesis	*atpA atpB atpE atpF^A^ atpH atpI*
Subunit of acetyl-CoA-carboxylase	Other genes	*accD*
c-type cytochrome synthesis gene	Other genes	*ccsA*
Envelop membrane protein	Other genes	*cemA*
ATP-dependent protease subunit p gene	Other genes	*clpP*^A^
Maturase	Other genes	*matK*
Subunits of NADH dehydrogenase	Genes for photosynthesis	*ndhA*^A^ *ndhB*^A,B^ *ndhC ndhD ndhE ndhF ndhG ndhH ndhI ndhJ ndhK*
Subunits of photosystem I	Genes for photosynthesis	*psaA psaB psaC psaI psaJ*
Subunits of photosystem II	Genes for photosynthesis	*psbA psbB psbC psbD psbE psbF psbH* *psbI psbJ psbK psbL psbM psbN psbT psbZ*
Subunits of cytochrome	Genes for photosynthesis	*petA petB*^A^ *petD*^A^ *petG petL petN*
Large subunit of Rubisco	Genes for photosynthesis	*rbcL*
Large subunit of ribosome	Self-replication	*rpl2*^B^ *rpl14 rpl16*^A^ *rpl20 rpl22 rpl23*^B^ *rpl32 rpl33 rpl36*
DNA dependent RNA polymerase	Self-replication	*rpoA rpoB rpoC1*^A^ *rpoC2*
Ribosomal RNA genes	Self-replication	*rrn16*^B^ *rrn23*^B^ *rrn4.5*^B^ *rrn5*^B^
Small subunit of ribosome	Self-replication	*rps2 rps3 rps4 rps7*^B^ *rps8 rps11 rps12*^A,B^ *rps14 rps15 rps16*^A^ *rps18 rps19*
Transfer RNA genes	Self-replication	*trnA-UGC*^A,B^ *trnC-GCA trnD-GUC trnE-UUC trnF-GAA trnfM-CAU trnG-UCC trnG-GCC trnH-GUG trnI-CAU*^B^ *trnI-GAU*^A,B^ *trnK-UUU*^A^ *trnL-CAA*^B^ *trnL-UAA*^A^ *trnL-UAG trnM-CAU trnN-GUU*^B^ *trnP-UGG trnQ-UUG trnR-ACG*^B^ *trnR-UCU trnS-GCU trnS-GGA trnS-UGA trnT-GGU trnT-UGU trnV-GAC*^B^ *trnV-UAC*^A^ *trnW-CCA trnY-GUA*
Conserved open reading frames	Genes of unknown function	*ycf1 ycf2*^B^ *ycf3*^A^ *ycf4*

A: Genes containing introns; B: Duplicated gene (Genes appear in the IR regions).

**Table 4:** The genes having intron in the *Heimia myrtifolia* chloroplast genome and the length of the exons and introns.

Gene	Location	ExonI (bp)	IntronI (bp)	ExonII (bp)	IntronII (bp)	ExonIII (bp)
*rps16*	LSC	224	861	40		
*rpoC1*	LSC	453	743	1608		
*atpF*	LSC	145	767	410		
*petB*	LSC	6	780	642		
*petD*	LSC	8	749	475		
*ndhB*	IR	756	685	777		
*ndhA*	SSC	540	1039	552		
*rpl16*	LSC	399	976	9		
rps12*	LSC	114		27	548	231
*ycf3*	LSC	153	796	230	756	124
*clpP*	LSC	228	585	292	836	71
*trnK-UUU*	LSC	35	2500	37		
*trnL-UAA*	LSC	37	532	50		
*trnV-UAC*	LSC	37	599	38		
*trnI-GAU*	IR	35	945	42		
*trnA-UGC*	IR	35	805	38		
*trnG-UCC*	LSC	23	727	52		

*rps12* gene is trans-spliced gene with the two duplicated 3’ end exons in IR regions and 5’ end exon in the LSC region.

**Table 5:** Base composition in the *Heimia myrtifolia* chloroplast genome.

	T	C	A	G	Length (bp)
Genome	31.9	18.8	31.1	18.2	159,219
LSC	33.2	17.9	31.8	17.1	88,571
SSC	34.6	16.2	34.9	14.4	18,822
IR	28.6	20.4	28.8	22.2	25,913
tRNA	22.8	26.8	23.9	26.6	2817
rRNA	19.9	25.1	24.9	30.1	9050
Protein-coding genes	32.1	19.4	30.2	18.4	81,047
1st position	31.5	18.7	34.6	17.6	27,010
2nd position	31.2	18.7	30.7	19.4	27,010
3rd position	33.5	23	25.2	18.2	27,010

## Supplementary Materials

Supplementary materials will be available online.

## References

[1] Gledhill D. (1989). The Names of Plants.

[2] Hegnauer R., Herfst A. (1958). Over *Heimia salicfolia* Link et Otto. Pharm. Weekbl..

[3] Malone M.H., Rother A. (1994). *Heimia salicifolia*: A phytochemical and phytopharmacologic review. J. Ethnopharmacol..

[4] Lema W.L., Blankenship J.W., Malone M.H. (1986). Prostaglandin synthetase inhibition by alkaloids of *Heimia salicifolia*. J. Ethnopharmacol..

[5] Liu J., Qi Z., Zhao Y., Fu C., Xiang Q.J. (2012). Molecular phylogenetics and evolution complete cpDNA genome sequence of *Smilax china* and phylogenetic placement of Liliales–Influences of gene partitions and taxon sampling. Mol. Phylogenet. Evol..

[6] Jansen R.K., Cai Z., Raubeson L.A., Daniell H., Depamphilis C.W., Leebens-Mack J., Müller K.F., Guisinger-Bellian M., Haberle R.C., Hansen A.K. (2007). Analysis of 81 genes from 64 plastid genomes resolves relationships in angiosperms and identifies genome-scale evolutionary patterns. Proc. Natl. Acad. Sci. USA.

[7] Wicke S., Schneeweiss G.M., DePamphilis C.W., Müller K.F., Quandt D. (2011). The evolution of the plastid chromosome in land plants: Gene content, gene order, gene function. Plant Mol. Biol..

[8] Asaf S., Waqas M., Khan A.L., Khan M.A., Kang S.M., Imran Q.M., Shahzad R., Bilal S., Yun B.W., Lee I.J. (2017). The complete chloroplast genome of wild rice (*Oryza minuta*) and its comparison to related species. Front. Plant Sci..

[9] Wang L., Wu Z.Q., Bystriakova N., Ansell S.W., Xiang Q.P., Heinrichs J., Scheider H., Zhang X.C. (2011). Phylogeography of the Sino-Himalayan Fern *Lepisorus clathratus* on “The Roof of the World”. PLoS ONE.

[10] Wang L., Scheider H., Wu Z.Q., He L.J., Zhang X.C., Xiang Q.P. (2012). *Indehiscent sporangia* enable the accumulation of local fern diversity at the Qinghai-Tibetan Plateau. BMC Evol. Biol..

[11] Wu Z.Q. (2016). The completed eight chloroplast genomes of tomato from *Solanum* genus. Mitochondrial DNA Part A.

[12] Wu Z.Q. (2016). The whole chloroplast genome of shrub willows (*Salix suchowensis*). Mitochondrial DNA Part A.

[13] Gu C.H., Tembrock L.R., Ohnson N.G., Simmons M.P., Wu Z.Q. (2016). The complete plastid genome of *Lagerstroemia fauriei* and loss of *rpl2* Intron from *Lagerstroemia* (Lythraceae). PLoS ONE.

[14] Li P., Zhang S., Li F., Zhang S., Zhang H., Wang X., Sun R., Bonnema G., Borm T.J. (2017). A phylogenetic analysis of chloroplast genomes elucidates the relationships of the six economically important *Brassica* species comprising the triangle of U. Front. Plant Sci..

[15] Niu Z., Xue Q., Zhu S., Sun J., Liu W., Ding X. (2017). The complete plastome sequences of four Orchid species: Insights into the evolution of the Orchidaceae and the utility of plastomic mutational hotspots. Front. Plant Sci..

[16] Wang Y., Zhan D.F., Jia X., Mei W.L., Dai H.F., Chen X.T., Peng S.Q. (2016). Complete chloroplast genome sequence of Aquilaria sinensis (Lour) Gilg and evolution analysis within the Malvales order. Front. Plant Sci..

[17] Yang Y., Zhou T., Yang J., Meng X., Zhu J., Zhao G. (2015). The complete chloroplast genome of *Quercus baronii* (*Quercus* L.). Mitochondrial DNA.

[18] Chaney L., Mangelson R., Ramaraj T., Jellen E.N., Maughan P.J. (2016). The complete chloroplast genome sequences for four *Amaranthus* species (Amaranthaceae). Appl. Plant Sci..

[19] Mallo D., Posada D. (2016). Multilocus inference of species trees and DNA barcoding. Philos. Trans. R. Soc. Lond. B Biol. Sci..

[20] Wu Z.Q., Tembrock L.R., Ge S. (2015). Are differences in genomic data sets due to true biological variants or errors in genome assembly: An example from two chloroplast genomes. PLoS ONE.

[21] Cauz-Santos L.A., Munhoz C.F., Rodde N., Cauet S., Santos A.A., Penha H.A., Dornelas M.C., Varani A.M., Oliveira G.C., Bergès H. (2017). The chloroplast genome of *Passiflora edulis* (Passifloraceae) sssembled from long sequence reads: Structural organization and phylogenomic studies in Malpighiales. Front. Plant Sci..

[22] Chen J., Hao Z., Xu H., Yang L., Liu G., Sheng Y., Zheng C., Zheng W., Cheng T., Shi J. (2015). The complete chloroplast genome sequence of the relict woody plant *Metasequoia glyptostroboides* Hu et Cheng. Front. Plant Sci..

[23] Nie X., Lv S., Zhang Y., Du X., Wang L., Biradar S.S., Tan X., Wan F., Weining S. (2012). Complete chloroplast genome sequence of a major invasive species, crofton weed (*Ageratina adenophora*). PLoS ONE.

[24] Ikemura T. (1981). Correlation between the abundance of *Escherichia coli* transfer RNAs and the occurrence of the respective codons in its protein genes: A proposal for a synonymous codon choice that is optimal for the *E. coli* translational system. J. Mol. Biol..

[25] Plotkin J.B., Kudla G. (2011). Synonymous but not the same: The causes and consequences of codon bias. Nat. Rev. Genet..

[26] Qi Y.Y., Xu W., Xing T., Zhao M.M., Li Y.L., Xia G.M., Wang M.C. (2015). Synonymous codon usage bias in the plastid genome is unrelated to gene structure and shows evolutionary heterogeneity. Evol. Bioinform..

[27] Raubeson L.A., Peery R., Chumley T.W., Dziubek C., Fourcade H.M., Boore J.L., Jansen R.K. (2007). Comparative chloroplast genomics: Analyses including new sequences from the angiosperms *Nuphar advena* and *Ranunculus macranthus*. BMC Genom..

[28] Qian J., Song J., Gao H., Zhu Y., Xu J., Pang X., Yao H., Sun C., Li X., Li C. (2013). The complete chloroplast genome sequence of the medicinal plant *Salvia miltiorrhiza*. PLoS ONE.

[29] Redwan R.M., Saidin A., Kumar S.V. (2015). Complete chloroplast genome sequence of MD-2 pineapple and its comparative analysis among nine other plants from the subclass Commelinidae. BMC Plant Biol..

[30] Kasiborski B.A., Bennett M.S., Linton E.W. (2016). The chloroplast genome of *Phacus orbicularis* (Euglenophyceae): An initial datum point for the phacaceae. J. Phycol..

[31] Kim K.J., Lee H.L. (2004). Complete chloroplast genome sequences from Korean ginseng (*Panax ginseng* Nees) and comparative analysis of sequence evolution among 17 vascular plants. DNA Res..

[32] Lu R.S., Li P., Qiu Y.X. (2017). The complete chloroplast genomes of three *Cardiocrinum* (Liliaceae) species: Comparative genomic and phylogenetic analyses. Front. Plant Sci..

[33] Ivanova Z., Sablok G., Daskalova E., Zahmanova G., Apostolova E., Yahubyan G., Baev V. (2017). Chloroplast genome analysis of resurrection tertiary relict *Haberlea rhodopensis* highlights genes important for desiccation stress response. Front. Plant Sci..

[34] Rubinsztein D.C., Amos W., Leggo J., Goodburn S., Jain S., Li S.H., Margolis R.L., Ross C.A., Ferguson-Smith M.A. (1995). Microsatellite evolution—Evidence for directionality and variation in rate between species. Nat. Genet..

[35] Gemayel R., Cho J., Boeynaems S., Verstrepen K.J. (2012). Beyond junk-variable tandem repeats as facilitators of rapid evolution of regulatory and coding sequences. Genes.

[36] Voronova A., Belevich V., Jansons A., Rungis D. (2014). Stress-induced transcriptional activation of retrotransposon-like sequences in the Scots pine (*Pinus sylvestris* L.) genome. Tree Genet. Genomes.

[37] Zhang Y., Du L., Liu A., Chen J., Wu L., Hu W., Zhang W., Lee S.C., Yang T.J., Wang Y. (2016). The complete chloroplast genome sequences of five *Epimedium* species: Lights into phylogenetic and taxonomic analyses. Front. Plant Sci..

[38] Zhuang Y., Tripp E.A. (2017). The draft genome of *Ruellia speciosa* (Beautiful Wild Petunia: Acanthaceae). DNA Res..

[39] Gu C.H., Tembrock L.R., Zheng S.Y., Wu Z.Q. (2018). The complete chloroplast genome of *Catha edulis*: A comparative analysis of genome features with related species. Int. J. Mol. Sci..

[40] Doyle J.J., Doyle J. (1987). A rapid DNA isolation procedure for small quantities of fresh leaf tissue. Phytochem. Bull..

[41] Bolger A.M., Lohse M., Usadel B. (2014). Trimmomatic: A flexible trimmer for Illumina sequence data. Bioinformatics.

[42] Wyman S.K., Jansen R.K., Boore J.L. (2004). Automatic annotation of organellar genomes with DOGMA. Bioinformatics.

[43] Schattner P., Brooks A.N., Lowe T.M. (2005). The tRNAscan-SE, snoscan and snoGPS web servers for the detection of tRNAs and snoRNAs. Nucleic Acids Res..

[44] Lohse M., Drechsel O., Bock R. (2007). OrganellarGenomeDRAW (OGDRAW): A tool for the easy generation of high-quality custom graphical maps of plastid and mitochondrial genomes. Curr. Genet..

[45] Tamura K., Stecher G., Peterson D., Filipski A., Kumar S. (2013). MEGA6: Molecular evolutionary genetics analysis version 6.0. Mol. Biol. Evol..

[46] Frazer K.A., Pachter L., Poliakov A., Rubin E.M., Dubchak I. (2004). VISTA: Computational tools for comparative genomics. Nucleic Acids Res..

[47] Rozas J., Sánchez-DelBarrio J.C., Messeguer X., Rozas R. (2003). DnaSP, DNA polymorphism analyses by the coalescent and other methods. Bioinformatics.

[48] Kurtz S., Choudhuri J.V., Ohlebusch E., Schleiermacher C., Stoye J., Giegerich R. (2001). REPuter: The manifold applications of repeat analysis on a genomic scale. Nucleic Acids Res..

[49] Li Q., Wan J.M. (2005). SSRHunter: Development of a local searching software for SSR sites. Yi Chuan.

[50] Simmons M.P., Cappa J.J., Archer R.H., Ford A.J., Eichstedt D., Clevinger C.C. (2008). Phylogeny of the Celastreae (Celastraceae) and the relationships of *Catha edulis* (qat) inferred from morphological characters and nuclear and plastid genes. Mol. Phylogenet. Evol..

[51] Guindon S., Dufayard J.F., Lefort V., Anisimova M. (2010). New algorithms and methods to estimate maximum-likelihoods phylogenies: Assessing the performance of PhyML 3.0. Syst. Biol..

[52] Ronquist F., Teslenko M., Van Der Mark P., Ayres D.L., Darling A., Höhna S., Larget B., Liu L., Suchard M.A., Huelsenbeck J.P. (2012). Mrbayes 3.2: Efficient bayesian phylogenetic inference and model choice across a large model space. Syst. Biol..

[53] Swofford D.L. (2002). Paup*: Phylogenetic analysis using parsimony (and other methods).

