# Supplementary material for: The Complete Chloroplast Genome of Heimia myrtifolia and Comparative Analysis within Myrtales
Source: Molecules. 2018 Apr 8;23(4):846. doi: 10.3390/molecules23040846 (PMC6017443; doi:10.3390/molecules23040846)
Supplement: Supplementary file 1 [file molecules-23-00846-s001.zip › Supplementary/Table S5 The GenBank accession numbers of 29 species using in phylogenetic analysis.docx]

Table S5 The GenBank accession numbers of 29 species using in phylogenetic analysis

| Number | Species | Genbank number |
| --- | --- | --- |
| 1 | *Acca sellowiana* | KX289887 |
| 2 | *Allosyncarpia ternata* | NC_022413 |
| 3 | *Angophora costata* | NC_022412 |
| 4 | *Angophora floribunda* | NC_022411 |
| 5 | *Corymbia eximia* | NC_022409 |
| 6 | *Corymbia gummifera* | NC_022407 |
| 7 | *Eucalyptus aromaphloia* | NC_022396 |
| 8 | *Eucalyptus baxteri* | NC_022382 |
| 9 | *Eugenia uniflora* | NC_027744 |
| 10 | *Erodium carvifolium* | NC_015083 |
| 11 | *Erodium crassifolium* | NC_025906 |
| 12 | *Geranium palmatum* | NC_014573 |
| 13 | *Heimia myrtifolia* | MG921615 |
| 14 | *Lagerstroemia fauriei* | NC_029808 |
| 15 | *Lagerstroemia floribunda* | NC_031825 |
| 16 | *Lagerstroemia guilinensis* | NC_029885 |
| 17 | *Lagerstroemia indica* | NC_030484 |
| 18 | *Lagerstroemia speciosa* | NC_031414 |
| 19 | *Lagerstroemia subcostata* | NC_034952 |
| 20 | *Lagerstroemia intermedia* | NC_034662 |
| 21 | *Ludwigia octovalvis* | NC_031385 |
| 22 | *Monsonia speciosa* | NC_014582 |
| 23 | *Oenothera argillicola* | NC_010358 |
| 24 | *Oenothera biennis* | NC_010361 |
| 25 | *Psidium guajava* | NC_033355 |
| 26 | *Pelargonium alternans* | NC_023261 |
| 27 | *Pelargonium x hortorum* | NC_008454 |
| 28 | *Stockwellia quadrifida* | NC_022414 |
| 29 | *Syzygium cumini* | GQ870669 |
